# Influence of *Escherichia coli* on Expression of Selected Human Drug Addiction Genes

**DOI:** 10.3390/life11121346

**Published:** 2021-12-05

**Authors:** Roman Kotłowski

**Affiliations:** Department of Molecular Biotechnology and Microbiology, Faculty of Chemistry, Gdansk University of Technology, Str. G. Narutowicza 11/12, 80-233 Gdańsk, Poland; roman.kotlowski@pg.edu.pl; Tel.: +48-5834-72383

**Keywords:** cocaine addiction, HT-29 cell line, RNA transcripts, pathogenic *Escherichia coli*

## Abstract

The impact of enteric microflora on the expression of genes associated with cocaine and amphetamine addiction was described. Human genome-wide experiments on RNA transcripts expressed in response to three selected *Escherichia coli* strains allowed for significant alteration (*p* > 0.05) of the linear regression model between HT-29 RNA transcripts associated with the KEGG pathway:hsa05030:Cocaine addiction after 3 h stimulation with intracellular pathogenic *E. coli* strain UM146 versus non-pathogenic *E. coli* Nissle 1917. Among the features influenced by the UM146 bacterial strain were visual learning, response to the presence of morphine, response to hypoxia, behavioral fear response and cognitive functions.

## 1. Introduction

The influence of *Escherichia coli* on the expression of drug addiction genes is a new subject when taking into account the genome-wide scale of experiments. There are generally two ways of addiction development described in relation to the impact of the gut microbiome or their metabolites on addiction as a consequence of substance use, and as a result of mediating behavioral response to drugs [1]. In the present study, the direct effect of *Escherichia coli* strains representing intracellular *E. coli* UM146 and extracellular *E. coli* UM147 pathogenic bacteria against the HT-29 cell line, in confrontation with non-pathogenic strain *E. coli* Nissle 1917, was studied. An original human genome-wide experiment using microarray was carried out for three *E. coli* strains in order to detect levels of RNA transcripts followed by grouping analysis of genes on the basis of molecular functions.

## 2. Materials and Methods

Two pathogenic strains, *E. coli* UM146 [2] and *E. coli* UM147, isolated from Crohn’s disease and ulcerative colitis cases, as well as non-pathogenic *E. coli* Nissle 1917, were applied in this study. We used selected bacteria for determination of the levels of RNA transcripts of human adenocarcinoma cells HT-29 in the genome-scale experiment following the Affymetrix^®^ guidance manual for expression microarray GeneChip HG-U133A Plus 2.0. The HT-29 cells were washed five times using physiological salt during exchanging the growth medium RPMI 1640 supplemented with 10% fetal bovine serum. The number of each *E. coli* strain applied for confluent HT-29 cells was ≈10^7^ suspended in 1 mL of Luria–Bertani liquid growth medium. The confluent HT-29 cells were present on 70% of the 75 cm^2^ surface of flat bottles. The incubation period of *E. coli* bacteria with confluent HT-29 cells lasted 3 h at 37 °C at normal atmosphere supplemented with 5% CO_2_. Microarray experiment was conducted only once without repetitions due to reproducibility problems of RNA fragmentation process using chemical method recommended by Affymetrix^®^ company. Statistical analysis for the linear regression model was performed using Winks SDA ver. 7.0.9 statistical software. Differentially expressed RNA transcripts of the HT-29 cell-line treated by *E. coli* were selected for standard deviation value SD ≥ 10% for the same ID probe signals, representing three different *E. coli* treatments of the HT-29 cell line. The maximum and minimum signal values including all microarray results were considered as 100% and 0%, respectively. Specificity of single-stranded microarray probes against human mRNAs was assessed by using salmon genetic material.

## 3. Results

Up to 187 ID probes specific to human RNA transcripts, differentially expressed, are presented in Supplementary Table S1. Gene ontology annotations analysis using the DAVID online tool [3] distinguished group no. 9, including genes related to drug addiction, listed in Table 1.

The complete list of human genes involved in addiction to cocaine and amphetamine based on the Kyoto Encyclopedia of Genes and Genomes (KEGG) is given in Table 2.

Statistical analysis revealed that in the case of intracellular pathogen *E. coli* UM146, isolated from a Crohn’s disease patient, there is significant change at *p*-value > 0.05 from the linear trend, as shown in Table 3, in comparison with *E. coli* UM147, an extracellular human pathogen isolated from an ulcerative colitis patient.

**Figure 1:**
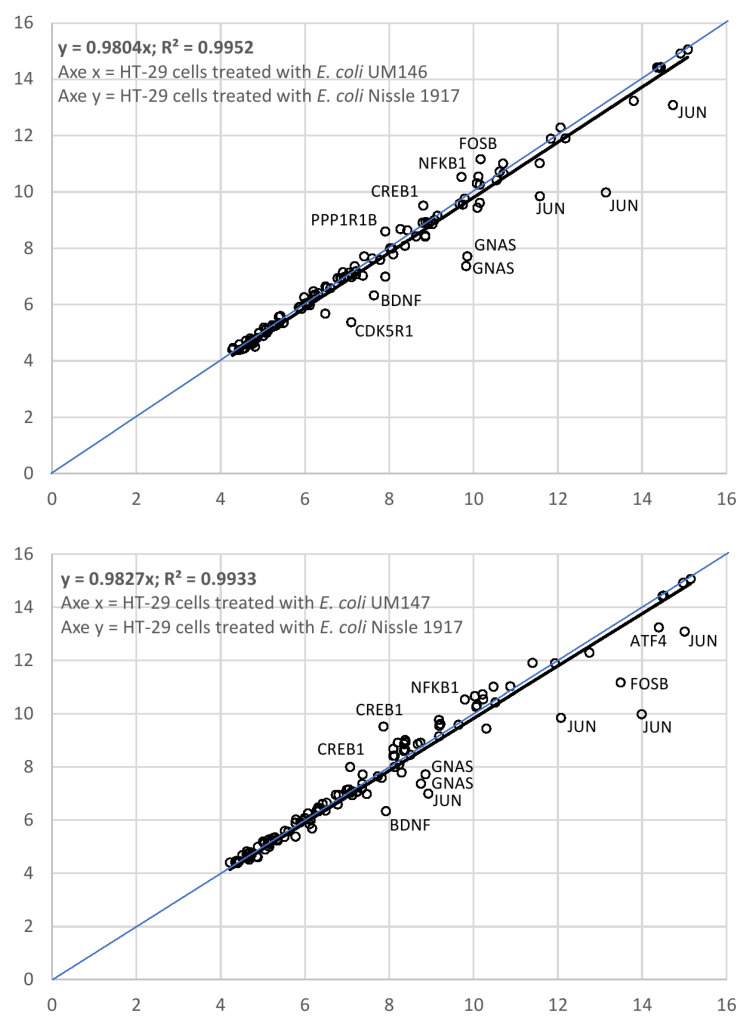
HT-29 cell RNA transcripts genes participated in KEGG pathway hsa05030:cocaine addiction.

Among up- and downregulated genes from the pathway hsa05030:cocaine addiction are genes JUN, GNAS, BDFN, and CDK5R1 (upregulated), and FOSB, NFKB1, CREB1 and PPP1R1B (downregulated). The multi-functional properties of selected genes are listed in Supplementary Table S2 based on information obtained from Affymetrix NetAffx™ Analysis Center (https://www.affymetrix.com/analysis/index.affx) for Batch Query option. Elevated transcription levels for human addiction genes to cocaine and amphetamine are indicated in Figure 1.

Among the up- and downregulated genes selected based on fluctuations from the linear regression model are the genes responsible for regulatory mechanisms, such as NFKB1, CREB1 and JUN, related to the transcription of genes. However, slightly downregulated PPP1R1B after only 3 h treatment of HT-29 cells with *E. coli* UM146 has direct implications for human perception, because this gene expression product functions as the D1 dopamine receptor binding molecule that is responsible for visual learning. Furthermore, the expression of the FOSB gene is also decreased for HT-29 cells stimulated by *E. coli* UM146 in comparison to the reference non-pathogenic *E. coli* strain and significantly decreased in relation to *E. coli* 147UM. This gene, among many functions, has a direct role in the response to the presence of morphine in humans, and its downregulation may also indicate a connection with the development of addiction to this drug. In addition, the determination of an increased level of the BDNF gene in our host–pathogen interactions model for two pathogenic *E. coli* strains indicates the response of HT-29 cells to hypoxia, most probably as a result of apoptosis induced by pathogenic bacteria. The same gene is also responsible for the behavioral fear response, suggesting by some means the necessity of drug intake. Interestingly, both pathogenic *E. coli* strains induced higher levels of RNA transcripts for the GNAS gene, which is responsible for cognition.

## 4. Discussion

The original results of our genomics analysis highlight the possibility of increasing the occurrence of addiction after infection of the GI tract by specific bacterial species. Differences in HT-29 cell responses to bacterial strains from the same species complicate understanding the mechanism of bacterial origin. However, the intracellular human pathogen *E. coli* UM146 has a stronger impact on the linear trend change than *E. coli* 147UM. Some conclusions can be drawn from the nature of host–microbial interaction. For example, strain *E. coli* UM146 [2] is an intracellular pathogen, while the other strain tested, *E. coli* UM147, does not show such features. Different levels of addiction genes may be related also to the presence of the PAI I genetic element present only in *E. coli* UM146. In conclusion, one of the most common bacterial species in the human gastrointestinal tract is responsible for the RNA transcript profiles of HT-29 cells related to cocaine addiction. Among three different *E. coli* strains used, the most influential in the present study model was intracellular pathogen *E. coli* 146UM. The presented results can give some clues for clinicians involved in the prescription of medicines linked with addiction potential for patients with accompanying enteric intracellular pathogen infections.

## Figures and Tables

**Table 1:** Enriched pathway proteins responding to infection with pathogenic *Escherichia coli* strains using DAVID online tool: https://david.ncifcrf.gov/.

Annotation Cluster 9	Enrichment Score: 1.2878	Count	%	*p*-Value	Genes
Category	Term				
GOTERM_BP_DIRECT	GO:0042493~response to drug	6	4.84	0.0314	JUN, CDKN1B, NDUFA10, GNAS, FOSB, ICAM1
KEGG_PATHWAY	hsa05030:cocaine addiction	3	2.42	0.0508	JUN, GNAS, FOSB
KEGG_PATHWAY	hsa05031:amphetamine addiction	3	2.42	0.0856	JUN, GNAS, FOSB

**Table 2:** List of human genes involved in addiction to cocaine, hsa05030:cocaine, and amphetamine, hsa05031:amphetamine.

Cocaine Addiction RNA Transcripts (*n* = 51)	Amphetamine Addiction RNA Transcripts (*n* = 71)
ADCY5 ^#^, ATF2, ATF4, ATF6B, BDNF, CDK5, DK5R1, CREB1, CREB3, CREB3L1, CREB3L2, CREB3L3, CREB3L4, CREB5, DDC, DLG4, DRD1, DRD2, FOSB, GNAI1, GNAI2, GNAI3, GNAS, GPSM1, GRIA2, GRIN1, GRIN2A, GRIN2B, GRIN2C, GRIN2D, GRIN3A, GRIN3B, GRM2, GRM3, JUN, LOC401317, MAOA, MAOB, NFKB1, PDYN, PPP1R1B, PRKACA, PRKACB, PRKACG, RELA, RGS9, SLC18A1, SLC18A2, SLC6A3, TH, TNXB	ADCY5, ARC, ATF2, ATF4, ATF6B, CACNA1C, CACNA1D, CALM1, CALM2, CALM3, CALML3, CALML4, CALML5, CALML6, CAMK2A, CAMK2B, CAMK2D, CAMK2G, CAMK4, CREB1, CREB3, CREB3L1, CREB3L2, CREB3L3, CREB3L4, CREB5, DDC, DRD1, FOS, FOSB, GNAS, GRIA1, GRIA2, GRIA3, GRIA4, GRIN1, GRIN2A, GRIN2B, GRIN2C, GRIN2D, GRIN3A, GRIN3B, HDAC1, HDAC2, JUN, LOC401317, MAOA, MAOB, PDYN, PPP1CA, PPP1CB, PPP1CC, PPP1R1B, PPP3CA, PPP3CB, PPP3CC, PPP3R1, PPP3R2, PRKACA, PRKACB, PRKACG, PRKCA, PRKCB, PRKCG, SIRT1, SLC18A1, SLC18A2, SLC6A3, STX1A, TH, TNXB

^#^ Common genes for both addiction types are highlighted in grey.

**Table 3:** Estimation of shift from linear regression of human RNA transcripts for genes from pathways: hsa05030:cocaine addiction and hsa05031:amphetamine addiction.

HT-29 Cell Line Responding to *E. coli*	Cocaine, *n* = 51		Amphetamine, *n* = 71	
	*t*-Test	*p*-Value (2 Tail)	*t*-Test	*p*-Value (2 Tail)
*E. coli* 146UM ver. *E. coli* Nissle 1917	1.976	*p* = 0.051	2.461	*p* = 0.015 *
*E. coli* 147UM ver. *E. coli* Nissle 1917	3.057	*p* = 0.03 *	2.928	*p* = 0.04 *

* Significant linear regression between RNA transcripts only for *p*-values < 0.05.

## Supplementary Materials

The following are available online at https://www.mdpi.com/article/10.3390/life11121346/s1, Table S1: The list of differentially expressed genes with the values of RNA transcripts read from microarray scanner, Table S2: Functions of selected differentially expressed RNA transcripts.
