# Supplementary material for: Influence of Escherichia coli on Expression of Selected Human Drug Addiction Genes
Source: Life (Basel). 2021 Dec 5;11(12):1346. doi: 10.3390/life11121346 (PMC8705772; doi:10.3390/life11121346)
Supplement: Supplementary file 1 [file life-11-01346-s001.zip › life-1497826-supplementary.pdf]

Supplementary Table S1. The list of differentially expressed genes with the values of RNA transcripts read from microarray scanner.

Microarray ID	*E. coli*_UM146.cel	*E. coli*_UM147.cel	*E. coli*_Nissle_1917.cel	*E. coli*_UM146.cel	*E. coli*_UM147.cel	*E. coli*_Nissle_1917.cel	SDEV
1559060_a_at	10.0058	10.7964	5.183	52.5051	59.1781	11.7988	25.6461
affx-m27830_5_at	15.5403	15.4968	10.3818	99.2184	98.8513	55.6787	25.0324
238355_at	9.556	9.561	4.8367	48.7086	48.7508	8.8759	23.0096
224566_at	13.1965	14.0459	8.9608	79.4358	86.6051	43.6849	22.9916
214657_s_at	9.2699	10.5102	5.7899	46.2938	56.7624	16.9213	20.6545
228999_at	11.9601	12.3449	8.0423	69.0002	72.2480	35.9324	20.0950
224565_at	13.6066	14.2093	10.0627	82.8972	87.9843	52.9854	18.9100
243206_at	8.6874	8.9664	5.1175	41.3773	43.7322	11.2460	18.1144
201170_s_at	9.5103	6.6097	10.7623	48.3229	23.8407	58.8903	17.9792
201465_s_at	13.1367	13.9886	9.985	78.9311	86.1215	52.3295	17.8009
1556814_a_at	8.7754	8.151	4.9609	42.1201	36.8499	9.9242	17.2691
1552329_at	9.6585	10.7034	6.7781	49.5738	58.3931	25.2621	17.1586
1557257_at	8.6467	8.262	4.9899	41.0338	37.7868	10.1690	16.9604
1558783_at	8.3703	7.8546	4.6693	38.7009	34.3481	7.4630	16.9192
202672_s_at	12.4646	13.9671	10.0428	73.2583	85.9400	52.8174	16.7121
213418_at	9.9926	8.9153	6.1664	52.3937	43.3009	20.0991	16.6530
1570585_at	8.6305	9.7863	5.9496	40.8970	50.6524	18.2692	16.6125
1555827_at	9.2827	9.8283	6.2009	46.4019	51.0069	20.3903	16.5085
228157_at	6.0452	5.5377	9.1193	19.0761	14.7926	45.0227	16.3576
243797_at	8.1045	8.3187	4.9287	36.4574	38.2653	9.6524	16.0233
229574_at	10.9029	10.7403	7.5709	60.0770	58.7046	31.9536	15.8557
207574_s_at	9.8939	11.797	8.086	51.5606	67.6235	36.3013	15.6629
232216_at	8.6952	9.0492	5.6892	41.4431	44.4310	16.0713	15.5827
1554420_at	8.4893	10.3987	6.7488	39.7053	55.8213	25.0148	15.4088
239404_at	9.6275	9.1895	6.2699	49.3121	45.6152	20.9727	15.4059
242431_at	8.2714	7.7281	4.8776	37.8661	33.2804	9.2211	15.3862
213593_s_at	9.8689	9.4769	6.5439	51.3496	48.0410	23.2853	15.3373

209383_at	11.9194	12.8565	9.3538	68.6566	76.5661	47.0020	15.3053
231989_s_at	10.319	10.7315	7.4304	55.1486	58.6303	30.7677	15.1815
223666_at	8.8625	10.1702	6.648	42.8552	53.8927	24.1640	15.0277
242865_at	9.1931	8.6736	5.883	45.6456	41.2608	17.7071	15.0253
1553338_at	8.5359	9.4955	6.0529	40.0986	48.1980	19.1411	14.9951
236948_x_at	7.7217	8.2732	4.9619	33.2264	37.8813	9.9326	14.9744
210764_s_at	10.1441	11.1962	7.743	53.6724	62.5525	33.4062	14.9392
243918_at	7.7019	8.6824	5.2588	33.0593	41.3351	12.4386	14.8813
213649_at	11.1977	12.8738	9.3647	62.5652	76.7121	47.0940	14.8140
244470_at	9.1562	8.1712	5.7506	45.3342	37.0204	16.5896	14.7918
207147_at	7.3321	8.164	4.8111	29.9380	36.9596	8.6598	14.7362
212966_at	10.5608	10.2743	7.4181	57.1895	54.7713	30.6639	14.6664
201289_at	11.1668	11.8297	8.5809	62.3044	67.8995	40.4784	14.4891
235716_at	10.6595	10.6379	7.6802	58.0226	57.8403	32.8761	14.4660
210077_s_at	8.0384	8.4889	5.3295	35.8995	39.7019	13.0353	14.4241
202768_at	10.1667	13.4885	11.1664	53.8632	81.9004	62.3010	14.3842
244803_at	9.3209	9.0196	6.241	46.7243	44.1812	20.7287	14.3309
1555847_a_at	9.8717	11.3296	7.9506	51.3733	63.6785	35.1584	14.3046
235984_at	9.1959	8.6934	6.0481	45.6692	41.4279	19.1006	14.2735
1565358_at	5.9924	9.3212	7.1399	18.6305	46.7268	28.3158	14.2722
1553736_at	8.1584	8.8088	5.612	36.9123	42.4020	15.4197	14.2601
1554980_a_at	9.5169	11.5316	8.1997	48.3786	65.3834	37.2609	14.1636
223797_at	8.7998	7.4585	5.4659	42.3260	31.0049	14.1866	14.1589
214016_s_at	11.7712	12.3605	9.2239	67.4058	72.3797	45.9056	14.0705
232392_at	11.1508	12.0233	8.8029	62.1693	69.5336	42.3522	14.0581
215029_at	8.1285	7.2822	4.9325	36.6600	29.5169	9.6845	13.9763
202638_s_at	9.2338	7.5601	10.8474	45.9891	31.8625	59.6085	13.8738
216279_at	7.1543	7.8639	4.7577	28.4373	34.4266	8.2091	13.7381
240231_at	9.0455	6.8329	5.8748	44.3998	25.7246	17.6379	13.7256
242146_at	9.9244	7.8605	6.7284	51.8181	34.3979	24.8426	13.6775

238988_at	9.6577	9.5446	6.8086	49.5670	48.6124	25.5195	13.6166
243985_at	8.9221	8.3645	5.8918	43.3583	38.6519	17.7814	13.6132
241843_at	8.442	8.1951	5.535	39.3060	37.2221	14.7698	13.6044
243683_at	7.6714	7.1473	4.6594	32.8019	28.3783	7.3794	13.5820
239451_at	9.7114	8.735	6.5682	50.0203	41.7791	23.4904	13.5783
214814_at	8.759	9.3344	6.3331	41.9816	46.8382	21.5061	13.4446
230494_at	9.1541	7.0966	6.0377	45.3164	27.9503	19.0128	13.3750
1558710_at	8.0097	8.2689	5.4174	35.6573	37.8450	13.7772	13.3090
218023_s_at	10.4306	11.9046	8.7813	56.0906	68.5317	42.1699	13.1878
220266_s_at	9.2728	12.0942	9.523	46.3183	70.1320	48.4301	13.1816
1559993_at	8.8981	8.3241	5.9629	43.1557	38.3109	18.3815	13.1302
225857_s_at	11.2763	12.78	9.6712	63.2286	75.9204	49.6810	13.1221
238738_at	9.2137	9.5169	6.6921	45.8195	48.3786	24.5362	13.0894
205767_at	11.4011	12.1581	9.178	64.2820	70.6713	45.5182	13.0741
65588_at	11.102	12.5939	9.509	61.7575	74.3497	48.3119	13.0212
affx-humrge/m10098_5_at	9.7535	10.7759	12.7687	50.3756	59.0050	75.8250	12.9425
202147_s_at	11.2886	12.5945	9.5472	63.3324	74.3547	48.6343	12.9039
209112_at	8.9975	7.8098	10.8419	43.9947	33.9700	59.5621	12.8957
1558975_at	6.9776	9.0857	6.1352	26.9459	44.7391	19.8358	12.8279
242558_at	9.1992	9.2721	6.6325	45.6971	46.3124	24.0332	12.6890
244841_at	9.2052	9.1893	6.5977	45.7477	45.6135	23.7394	12.6679
244185_at	9.2645	8.976	6.5358	46.2482	43.8132	23.2170	12.6529
1553962_s_at	9.1423	10.8723	7.8939	45.2168	59.8187	34.6799	12.6241
212964_at	9.1254	8.8912	6.4325	45.0742	43.0975	22.3451	12.5909
228098_s_at	12.7686	12.9026	10.2547	75.8242	76.9552	54.6059	12.5896
1558976_x_at	7.1086	9.2105	6.3619	28.0516	45.7925	21.7492	12.4669
242725_at	7.9978	6.2983	9.2339	35.5568	21.2124	45.9900	12.4401
209305_s_at	8.3709	10.2328	7.3231	38.7059	54.4211	29.8621	12.4387
241425_at	8.631	6.6314	5.7576	40.9013	24.0239	16.6487	12.4327
243857_at	9.2221	8.4729	6.3876	45.8904	39.5668	21.9661	12.3972

214169_at	8.1318	9.4061	6.4903	36.6878	47.4434	22.8329	12.3377
205476_at	12.2577	10.1078	12.8919	71.5120	53.3660	76.8649	12.3162
223774_at	10.3779	12.2967	9.4412	55.6458	71.8412	47.7397	12.2860
239436_at	7.7621	7.6395	5.1849	33.5674	32.5326	11.8149	12.2710
239937_at	8.5202	9.5994	6.7313	39.9661	49.0749	24.8671	12.2268
225786_at	6.6372	5.7508	8.5785	24.0728	16.5913	40.4581	12.2071
229450_at	7.1391	6.1476	8.9927	28.3091	19.9404	43.9542	12.1892
242376_at	7.7209	8.3971	5.6281	33.2197	38.9271	15.5556	12.1848
209304_x_at	9.3835	11.318	8.5067	47.2527	63.5806	39.8521	12.1409
1555411_a_at	12.8104	13.9782	11.118	76.1770	86.0337	61.8925	12.1381
219312_s_at	9.2052	8.9126	6.5834	45.7477	43.2781	23.6187	12.1263
1559490_at	7.1555	7.247	4.7143	28.4475	29.2198	7.8428	12.1252
223780_s_at	7.8351	8.9482	6.1309	34.1836	43.5786	19.7995	11.9765
242688_at	9.7797	9.4983	7.2075	50.5967	48.2216	28.8864	11.9082
213326_at	5.9472	7.809	5.0625	18.2490	33.9633	10.7817	11.8327
214942_at	6.0542	7.5084	4.7228	19.1521	31.4261	7.9145	11.7596
226370_at	8.7598	9.0866	6.535	41.9884	44.7467	23.2102	11.7193
212225_at	9.1421	9.0338	6.694	45.2151	44.3011	24.5522	11.6748
241824_at	5.9822	7.1689	4.4115	18.5444	28.5606	5.2871	11.6743
230123_at	8.3863	9.1738	6.4912	38.8359	45.4827	22.8405	11.6383
1554719_at	6.7071	8.0687	5.3139	24.6628	36.1552	12.9037	11.6260
230733_at	5.4393	5.0407	7.5905	13.9621	10.5977	32.1190	11.5770
241495_at	9.0635	9.0977	6.7091	44.5517	44.8404	24.6797	11.5574
1568627_at	7.8602	9.062	6.3465	34.3954	44.5391	21.6192	11.4851
243543_at	8.4747	6.2725	5.9962	39.5820	20.9946	18.6625	11.4641
222872_x_at	10.3118	9.9157	7.7908	55.0879	51.7446	33.8097	11.4426
220319_s_at	10.6326	11.5436	8.886	57.7955	65.4847	43.0536	11.3989
239203_at	6.4581	8.5726	6.0678	22.5612	40.4083	19.2669	11.3749
affx-m27830_m_at	13.7023	13.7764	11.4063	83.7050	84.3304	64.3259	11.3734
1557352_at	10.3239	8.4181	7.7209	55.1900	39.1043	33.2197	11.3730

1554602_at	8.0576	7.93	5.6677	36.0615	34.9846	15.8899	11.3480
203395_s_at	9.4194	8.3144	10.9798	47.5557	38.2290	60.7260	11.3031
1568695_s_at	7.7309	8.8768	6.2138	33.3041	42.9759	20.4992	11.2747
225239_at	10.1331	11.101	8.4693	53.5796	61.7490	39.5365	11.2350
214683_s_at	11.2035	12.3193	9.6698	62.6142	72.0319	49.6691	11.2277
232141_at	9.5865	10.5505	7.9281	48.9661	57.1026	34.9685	11.1956
205239_at	12.9125	13.7569	11.1628	77.0388	84.1658	62.2706	11.1676
202014_at	11.073	12.5164	9.8742	61.5127	73.6955	51.3944	11.1665
236699_at	10.7389	10.7983	8.4792	58.6928	59.1941	39.6200	11.1592
241786_at	7.6124	4.9709	6.3096	32.3039	10.0086	21.3078	11.1480
228346_at	7.0485	8.8804	6.3191	27.5444	43.0063	21.3879	11.1380
202146_at	11.2073	12.3376	9.7141	62.6462	72.1864	50.0430	11.1069
233085_s_at	9.4138	8.9491	6.945	47.5084	43.5862	26.6708	11.0734
1560274_at	7.6776	6.8677	5.1114	32.8542	26.0183	11.1945	11.0726
232696_at	7.5322	7.6925	5.3532	31.6270	32.9800	13.2354	11.0297
204025_s_at	8.7188	6.8813	9.4085	41.6423	26.1331	47.4637	11.0258
213575_at	8.9715	8.6391	6.5655	43.7752	40.9696	23.4676	11.0045
1556060_a_at	11.028	10.3089	8.4991	61.1329	55.0634	39.7880	10.9983
228846_at	11.033	11.2121	8.8731	61.1751	62.6867	42.9447	10.9877
239757_at	9.7471	8.5826	7.1596	50.3216	40.4927	28.4821	10.9379
223773_s_at	11.6824	13.487	10.9759	66.6563	81.8878	60.6931	10.9299
232865_at	8.657	8.147	6.2077	41.1207	36.8161	20.4477	10.9074
207980_s_at	6.1623	8.0651	8.6257	20.0645	36.1249	40.8565	10.8983
243296_at	8.6397	6.5693	6.2692	40.9747	23.4997	20.9668	10.8942
209446_s_at	6.7931	5.6648	8.2361	25.3887	15.8654	37.5682	10.8784
240452_at	9.0025	6.5176	7.1735	44.0369	23.0634	28.5994	10.8693
208118_x_at	9.5186	10.1641	7.6869	48.3930	53.8412	32.9327	10.8464
223129_x_at	11.6653	12.4087	9.9066	66.5119	72.7865	51.6678	10.8453
202388_at	11.5776	12.8871	10.3176	65.7717	76.8244	55.1368	10.8445
1561817_at	7.7536	6.6199	5.1936	33.4957	23.9268	11.8883	10.8272

225699_at	11.7215	13.0109	10.4478	66.9863	77.8693	56.2358	10.8168
1554229_at	9.9612	9.9407	7.7354	52.1287	51.9556	33.3421	10.7969
222802_at	8.6585	8.5733	10.8266	41.1334	40.4143	59.4330	10.7789
224454_at	8.3378	8.7158	6.3396	38.4265	41.6170	21.5610	10.7771
212514_x_at	11.3909	12.0774	9.6063	64.1959	69.9902	49.1332	10.7663
202149_at	6.9776	6.7748	9.0757	26.9459	25.2342	44.6547	10.7524
242836_at	7.0722	5.4496	4.5611	27.7444	14.0490	6.5497	10.7472
220046_s_at	12.8742	13.7328	11.2391	76.7155	83.9624	62.9146	10.6926
233899_x_at	8.6842	8.1818	6.2879	41.3503	37.1098	21.1246	10.6661
202301_s_at	11.0913	11.9081	9.4285	61.6671	68.5613	47.6325	10.6655
236241_at	8.6966	8.5277	6.4392	41.4550	40.0294	22.4016	10.6129
201044_x_at	10.138	10.7314	8.3187	53.6209	58.6295	38.2653	10.6111
218566_s_at	11.1728	11.8879	9.4442	62.3550	68.3908	47.7650	10.6044
37028_at	11.5959	13.0228	10.5205	65.9262	77.9697	56.8494	10.5949
239405_at	7.3954	7.296	5.1902	30.4723	29.6333	11.8596	10.5123
228097_at	9.6511	9.3638	7.365	49.5113	47.0864	30.2157	10.5105
1554014_at	9.9527	10.237	7.9562	52.0569	54.4565	35.2057	10.4906
223130_s_at	11.1555	12.4553	9.9723	62.2090	73.1798	52.2224	10.4826
237180_at	8.4414	7.0234	5.9675	39.3010	27.3325	18.4203	10.4775
236480_at	4.9993	5.0161	7.1426	10.2483	10.3901	28.3386	10.4037
228238_at	10.6843	10.9394	8.6951	58.2319	60.3851	41.4423	10.3711
229274_at	9.8192	8.7494	7.3695	50.9301	41.9006	30.2537	10.3658
1559391_s_at	8.1282	5.6863	7.1245	36.6574	16.0469	28.1858	10.3595
1556602_at	7.8234	7.1624	5.4468	34.0848	28.5057	14.0254	10.3536
209259_s_at	9.2685	7.965	10.4084	46.2820	35.2800	55.9032	10.3193
211721_s_at	8.7517	8.4477	6.4992	41.9200	39.3541	22.9081	10.3159
204109_s_at	9.2734	9.3562	7.2138	46.3234	47.0222	28.9395	10.2443
230097_at	9.931	8.6575	7.5048	51.8738	41.1249	31.3957	10.2433
213700_s_at	7.2029	6.638	8.9613	28.8475	24.0796	43.6891	10.2269
223394_at	11.4387	11.7436	9.5108	64.5993	67.1728	48.3271	10.2190

234989_at	9.3113	10.5797	8.161	46.6433	57.3490	36.9343	10.2114
221841_s_at	11.7976	13.656	11.3952	67.6286	83.3142	64.2322	10.1792
200664_s_at	11.715	11.5771	9.5651	66.9314	65.7675	48.7854	10.1573
1565651_at	9.755	9.6875	7.6436	50.3883	49.8185	32.5672	10.1285
244727_at	7.7187	9.1893	6.8175	33.2011	45.6135	25.5946	10.1051
218689_at	8.4731	7.6347	9.9935	39.5685	32.4921	52.4013	10.0923
212099_at	12.698	14.3169	11.9832	75.2283	88.8925	69.1951	10.0920
206336_at	5.0784	4.579	6.8441	10.9160	6.7008	25.8191	10.0448
224559_at	10.592	10.4805	8.4806	57.4529	56.5118	39.6318	10.0283
202637_s_at	8.9702	7.7649	10.1347	43.7642	33.5910	53.5931	10.0015

In background-colored columns are %-RNA transcripts values in relation to all microarray results, after taking into account maximal and minimal results used as referential values of 100% and 0%, respectively.

Supplementary Table S2. Functions of selected differentially expressed RNA transcripts.

Gene Title	Gene Symbol	go biological process term	go molecular function term	go cellular component term	Pathway
jun proto-oncogene	JUN	angiogenesis	RNA polymerase II core promoter proximal region sequence-specific DNA binding	nuclear chromosome	
		microglial cell activation	RNA polymerase II distal enhancer sequence-specific DNA binding	nucleus	
		release of cytochrome c from mitochondria	RNA polymerase II transcription factor activity, sequence-specific DNA binding	nucleoplasm	
		liver development	transcriptional activator activity, RNA polymerase II core promoter proximal region sequence-specific binding	transcription factor complex	
		positive regulation of endothelial cell proliferation	RNA polymerase II activating transcription factor binding	nuclear euchromatin	
		toll-like receptor signaling pathway	transcriptional activator activity, RNA polymerase II transcription factor binding	cytosol	

Gene Title	Gene Symbol	go biological process term	go molecular function term	go cellular component term	Pathway
		MyD88-dependent toll-like receptor signaling pathway	DNA binding	transcriptional repressor complex	
		MyD88-independent toll-like receptor signaling pathway	chromatin binding		
		outflow tract morphogenesis	transcription factor activity, sequence-specific DNA binding		
		transcription from RNA polymerase II promoter	transcription factor activity, RNA polymerase II distal enhancer sequence-specific binding		
		transforming growth factor beta receptor signaling pathway	transcription coactivator activity		
		SMAD protein import into nucleus	GTPase activator activity		
		aging	protein binding		
		learning	transcription factor binding		
		circadian rhythm	enzyme binding		
		negative regulation of cell proliferation	cAMP response element binding		
		response to radiation	transcription regulatory region DNA binding		
		response to mechanical stimulus	poly(A) RNA binding		
		regulation of cell death	R-SMAD binding		
		monocyte differentiation	HMG box domain binding		
		axon regeneration			
		negative regulation of protein autophosphorylation			
		response to lipopolysaccharide			

Gene Title	Gene Symbol	go biological process term	go molecular function term	go cellular component term	Pathway
		cellular response to hormone stimulus			
		response to cytokine			
		toll-like receptor 2 signaling pathway			
		toll-like receptor 3 signaling pathway			
		toll-like receptor 4 signaling pathway			
		toll-like receptor 5 signaling pathway			
		toll-like receptor 9 signaling pathway			
		toll-like receptor 10 signaling pathway			
		leading edge cell differentiation			
		TRIF-dependent toll-like receptor signaling pathway			
		response to muscle stretch			
		Fc-epsilon receptor signaling pathway			
		toll-like receptor TLR1:TLR2 signaling pathway			
		toll-like receptor TLR6:TLR2 signaling pathway			
		regulation of cell proliferation			
		response to drug			
		response to hydrogen peroxide			
		negative regulation of DNA binding			
		negative regulation of neuron apoptotic process			
		positive regulation of neuron apoptotic process			

Gene Title	Gene Symbol	go biological process term	go molecular function term	go cellular component term	Pathway
		positive regulation of GTPase activity			
		negative regulation by host of viral transcription			
		positive regulation by host of viral transcription			
		innate immune response			
		positive regulation of cell differentiation			
		positive regulation of monocyte differentiation			
		positive regulation of DNA replication			
		negative regulation of transcription, DNA-templated			
		positive regulation of transcription, DNA-templated			
		positive regulation of transcription from RNA polymerase II promoter			
		positive regulation of fibroblast proliferation			
		positive regulation of smooth muscle cell proliferation			
		regulation of sequence-specific DNA binding transcription factor activity			
		cellular response to potassium ion starvation			
		stress-activated MAPK cascade			
		response to cAMP			
		regulation of cell cycle			
		membrane depolarization			
		SMAD protein signal transduction			

Gene Title	Gene Symbol	go biological process term	go molecular function term	go cellular component term	Pathway
		cellular response to calcium ion			
		positive regulation of pri-miRNA transcription from RNA polymerase II promoter			
		negative regulation of transcription from RNA polymerase II promoter in response to endoplasmic reticulum stress			
		positive regulation of DNA-templated transcription, initiation			
GNAS complex locus	GNAS	skeletal system development	nucleotide binding	ruffle	
		tissue homeostasis	GTPase activity	extracellular region	
		endochondral ossification	adenylate cyclase activity	nucleus	
		renal water homeostasis	signal transducer activity	cytoplasm	
		energy reserve metabolic process	insulin-like growth factor receptor binding	cytosol	
		cAMP biosynthetic process	protein binding	heterotrimeric G-protein complex	
		DNA methylation	GTP binding	plasma membrane	
		water transport	guanyl nucleotide binding	membrane	
		signal transduction	G-protein beta/gamma-subunit complex binding	transport vesicle	
		G-protein coupled receptor signaling pathway	beta-2 adrenergic receptor binding	dendrite	
		adenylate cyclase-activating G-protein coupled receptor signaling pathway	D1 dopamine receptor binding	intrinsic component of membrane	
		activation of adenylate cyclase activity	mu-type opioid receptor binding	cytoplasmic vesicle	

Gene Title	Gene Symbol	go biological process term	go molecular function term	go cellular component term	Pathway
		adenylate cyclase-activating dopamine receptor signaling pathway	ionotropic glutamate receptor binding	trans-Golgi network membrane	
		female pregnancy	metal ion binding	cell projection	
		blood coagulation	corticotropin-releasing hormone receptor 1 binding	perinuclear region of cytoplasm	
		sensory perception of chemical stimulus		extracellular exosome	
		sensory perception of smell			
		metabolic process			
		protein secretion			
		post-embryonic development			
		regulation of signal transduction			
		positive regulation of cAMP biosynthetic process			
		embryonic hindlimb morphogenesis			
		multicellular organism growth			
		negative regulation of multicellular organism growth			
		post-embryonic body morphogenesis			
		response to drug			
		positive regulation of GTPase activity			
		skin development			
		positive regulation of cAMP-mediated signaling			

Gene Title	Gene Symbol	go biological process term	go molecular function term	go cellular component term	Pathway
		small molecule metabolic process			
		positive regulation of osteoblast differentiation			
		positive regulation of osteoclast differentiation			
		intracellular transport			
		developmental growth			
		embryonic cranial skeleton morphogenesis			
		regulation of insulin secretion			
		cognition			
		cartilage development			
		transmembrane transport			
		bone development			
		hair follicle placode formation			
		platelet aggregation			
		response to parathyroid hormone			
		cellular response to glucagon stimulus			
		cellular response to prostaglandin E stimulus			
		genetic imprinting			
		cellular response to catecholamine stimulus			
		adenylate cyclase-activating adrenergic receptor signaling pathway			

Gene Title	Gene Symbol	go biological process term	go molecular function term	go cellular component term	Pathway
brain-derived neurotrophic factor	BDNF	ureteric bud development	receptor binding	extracellular region	
		behavioral fear response	neurotrophin TRKB receptor binding	extracellular space	
		response to hypoxia	protein binding	cytoplasm	
		chronic inflammatory response	growth factor activity	synaptic vesicle	
		mitochondrial electron transport, NADH to ubiquinone		membrane	
		transmembrane receptor protein tyrosine kinase signaling pathway		integral component of membrane	
		gamma-aminobutyric acid signaling pathway		cytoplasmic membrane-bounded vesicle	
		cell-cell signaling		mitochondrial crista	
		nervous system development		axon	
		negative regulation of neuroblast proliferation		dendrite	
		axon guidance		neuronal cell body	
		axon target recognition		terminal bouton	
		synapse assembly		perikaryon	
		learning or memory		perinuclear region of cytoplasm	
		learning			
		circadian rhythm			
		feeding behavior			

Gene Title	Gene Symbol	go biological process term	go molecular function term	go cellular component term	Pathway
		neuron recognition			
		response to light stimulus			
		response to light intensity			
		response to hormone			
		response to auditory stimulus			
		glutamate secretion			
		response to fluoxetine			
		response to activity			
		dendrite development			
		regulation of metabolic process			
		nerve development			
		regulation of axon extension			
		brain-derived neurotrophic factor receptor signaling pathway			
		positive regulation of brain-derived neurotrophic factor receptor signaling pathway			
		response to nutrient levels			
		response to food			
		negative regulation of synaptic transmission, GABAergic			
		positive regulation of peptidyl-serine phosphorylation			
		response to vitamin A			

Gene Title	Gene Symbol	go biological process term	go molecular function term	go cellular component term	Pathway
		response to tumor necrosis factor			
		response to potassium ion			
		mechanoreceptor differentiation			
		response to drug			
		fear response			
		negative regulation of apoptotic process			
		regulation of neuron apoptotic process			
		negative regulation of neuron apoptotic process			
		regulation of neuron differentiation			
		positive regulation of neuron differentiation			
		negative regulation of striated muscle tissue development			
		regulation of retinal cell programmed cell death			
		regulation of synaptic plasticity			
		regulation of long-term neuronal synaptic plasticity			
		positive regulation of long-term neuronal synaptic plasticity			
		regulation of short-term neuronal synaptic plasticity			
		collateral sprouting			
		positive regulation of collateral sprouting			
		axon extension			

Gene Title	Gene Symbol	go biological process term	go molecular function term	go cellular component term	Pathway
		neuron projection morphogenesis			
		inner ear development			
		cognition			
		positive regulation of sequence-specific DNA binding transcription factor activity			
		response to electrical stimulus			
		positive regulation of synapse assembly			
		response to hyperoxia			
		excitatory postsynaptic potential			
		inhibitory postsynaptic potential			
		negative regulation of cell death			
		taste bud development			
		cellular response to tumor necrosis factor			
		cellular response to norepinephrine stimulus			
		response to anesthetic			
		dendrite extension			
		negative regulation of neuron death			
		response to L-glutamate			
		response to nerve growth factor			
		cellular response to nerve growth factor stimulus			
		neuron projection extension			

Gene Title	Gene Symbol	go biological process term	go molecular function term	go cellular component term	Pathway
		positive regulation of glucocorticoid receptor signaling pathway			
cyclin-dependent kinase 5, regulatory subunit 1 (p35)	CDK5R1	regulation of cyclin-dependent protein serine/threonine kinase activity	protein kinase activity	nucleus	
		neuron migration	cyclin-dependent protein serine/threonine kinase activity	nucleoplasm	
		protein phosphorylation	calcium ion binding	cytoplasm	
		neuron cell-cell adhesion	protein binding	cytosol	
		G-protein coupled acetylcholine receptor signaling pathway	kinase activity	plasma membrane	
		axon guidance	cyclin-dependent protein kinase 5 activator activity	postsynaptic density	
		axonal fasciculation	protein kinase binding	membrane	
		brain development	protein serine/threonine kinase activator activity	cyclin-dependent protein kinase 5 holoenzyme complex	
		cell proliferation	cadherin binding	axon	
		embryo development	ephrin receptor binding	dendrite	
		phosphorylation		growth cone	
		peptidyl-serine phosphorylation		neuromuscular junction	
		peptidyl-threonine phosphorylation		neuronal cell body	
		cerebellum development		dendritic spine	
		superior olivary nucleus maturation		intracellular membrane-	

Gene Title	Gene Symbol	go biological process term	go molecular function term	go cellular component term	Pathway
				bounded organelle	
		hippocampus development		contractile fiber	
		cerebral cortex radially oriented cell migration		perinuclear region of cytoplasm	
		layer formation in cerebral cortex			
		neuron differentiation			
		negative regulation of axon extension			
		neuron projection development			
		regulation of actin cytoskeleton organization			
		serine phosphorylation of STAT3 protein			
		ionotropic glutamate receptor signaling pathway			
		positive regulation of neuron apoptotic process			
		regulation of neuron differentiation			
		positive regulation of protein kinase activity			
		negative regulation of transcription, DNA-templated			
		ephrin receptor signaling pathway			
		rhythmic process			
		regulation of cell cycle			
		regulation of dendritic spine morphogenesis			
		regulation of microtubule cytoskeleton organization			

Gene Title	Gene Symbol	go biological process term	go molecular function term	go cellular component term	Pathway
		positive regulation of cell cycle arrest			
		positive regulation of protein serine/threonine kinase activity			
		positive regulation of protein targeting to membrane			
FBJ murine osteosarcoma viral oncogene homolog B	FOSB	negative regulation of transcription from RNA polymerase II promoter	RNA polymerase II core promoter proximal region sequence-specific DNA binding	nucleus	
		regulation of transcription, DNA-templated	transcription factor activity, RNA polymerase II core promoter proximal region sequence-specific binding		
		regulation of transcription from RNA polymerase II promoter	transcriptional activator activity, RNA polymerase II core promoter proximal region sequence-specific binding		
		transcription from RNA polymerase II promoter	DNA binding		
		female pregnancy	double-stranded DNA binding		
		response to mechanical stimulus	transcription factor activity, sequence-specific DNA binding		
		response to progesterone	transcription factor binding		
		cellular response to hormone stimulus	sequence-specific DNA binding		
		response to drug			
		response to morphine			
		positive regulation of transcription from RNA polymerase II promoter			
		response to corticosterone			
		response to cAMP			

Gene Title	Gene Symbol	go biological process term	go molecular function term	go cellular component term	Pathway
		cellular response to calcium ion			
nuclear factor of kappa light polypeptide gene enhancer in B-cells 1	NFKB1	negative regulation of transcription from RNA polymerase II promoter	regulatory region DNA binding	nucleus	Apoptosis
		negative regulation of cytokine production	transcription regulatory region sequence-specific DNA binding	nucleoplasm	Apoptosis_GenMAPP
		stimulatory C-type lectin receptor signaling pathway	RNA polymerase II regulatory region sequence-specific DNA binding	cytoplasm	Apoptosis_KEGG
		toll-like receptor signaling pathway	RNA polymerase II distal enhancer sequence-specific DNA binding	mitochondrion	Smooth_muscle_contraction
		MyD88-dependent toll-like receptor signaling pathway	transcriptional activator activity, RNA polymerase II distal enhancer sequence-specific binding	cytosol	TGF_Beta_Signaling_Pathway
		MyD88-independent toll-like receptor signaling pathway	transcriptional repressor activity, RNA polymerase II transcription regulatory region sequence-specific binding	I-kappaB/NF-kappaB complex	
		transcription, DNA-templated	DNA binding	protein complex	
		regulation of transcription, DNA-templated	chromatin binding		
		regulation of transcription from RNA polymerase II promoter	double-stranded DNA binding		
		transcription from RNA polymerase II promoter	transcription factor activity, sequence-specific DNA binding		
		apoptotic process	protein binding		
		inflammatory response	transcription factor binding		
		response to oxidative stress	heat shock protein binding		
		signal transduction	identical protein binding		

Gene Title	Gene Symbol	go biological process term	go molecular function term	go cellular component term	Pathway
		I-kappaB kinase/NF-kappaB signaling	protein homodimerization activity		
		response to bacterium	actinin binding		
		positive regulation of macrophage derived foam cell differentiation	sequence-specific DNA binding		
		positive regulation of lipid storage	transcription regulatory region DNA binding		
		negative regulation of calcidiol 1-monooxygenase activity	protein heterodimerization activity		
		negative regulation of vitamin D biosynthetic process			
		membrane protein intracellular domain proteolysis			
		negative regulation of cellular protein metabolic process			
		negative regulation of cholesterol transport			
		positive regulation of type I interferon production			
		toll-like receptor 2 signaling pathway			
		toll-like receptor 3 signaling pathway			
		toll-like receptor 4 signaling pathway			
		toll-like receptor 5 signaling pathway			
		toll-like receptor 9 signaling pathway			
		toll-like receptor 10 signaling pathway			
		TRIF-dependent toll-like receptor signaling pathway			
		response to muscle stretch			
		NIK/NF-kappaB signaling			
		Fc-epsilon receptor signaling pathway			
		toll-like receptor TLR1:TLR2 signaling pathway			
		toll-like receptor TLR6:TLR2 signaling pathway			
		negative regulation of apoptotic process			

Gene Title	Gene Symbol	go biological process term	go molecular function term	go cellular component term	Pathway
		negative regulation of interleukin-12 biosynthetic process			
		innate immune response			
		negative regulation of transcription, DNA-templated			
		positive regulation of transcription, DNA-templated			
		positive regulation of transcription from RNA polymerase II promoter			
		response to copper ion			
		neurotrophin TRK receptor signaling pathway			
		negative regulation of inflammatory response			
		T cell receptor signaling pathway			
		positive regulation of NF-kappaB transcription factor activity			
		stress-activated MAPK cascade			
		cellular response to lipopolysaccharide			
		cellular response to mechanical stimulus			
		cellular response to nicotine			
		cellular response to interleukin-1			
		cellular response to interleukin-6			
		cellular response to dsRNA			
		cellular response to peptide hormone stimulus			
		positive regulation of canonical Wnt signaling pathway			
		positive regulation of hyaluronan biosynthetic process			
		positive regulation of miRNA metabolic process			
cAMP responsive element	CREB1	toll-like receptor signaling pathway	RNA polymerase II core promoter proximal region sequence-specific DNA binding	chromatin	

Gene Title	Gene Symbol	go biological process term	go molecular function term	go cellular component term	Pathway
binding protein 1					
		MyD88-dependent toll-like receptor signaling pathway	RNA polymerase II distal enhancer sequence-specific DNA binding	nuclear chromatin	
		MyD88-independent toll-like receptor signaling pathway	transcriptional activator activity, RNA polymerase II core promoter proximal region sequence-specific binding	nucleus	
		transcription, DNA-templated	RNA polymerase II activating transcription factor binding	nucleoplasm	
		regulation of transcription, DNA-templated	transcriptional activator activity, RNA polymerase II transcription factor binding	transcription factor complex	
		transcription from RNA polymerase II promoter	DNA binding	nuclear euchromatin	
		protein phosphorylation	double-stranded DNA binding	mitochondrion	
		signal transduction	transcription factor activity, sequence-specific DNA binding	ATF4-CREB1 transcription factor complex	
		epidermal growth factor receptor signaling pathway	transcription factor activity, RNA polymerase II distal enhancer sequence-specific binding		
		transforming growth factor beta receptor signaling pathway	transcription cofactor activity		
		activation of phospholipase C activity	protein binding		
		Notch signaling pathway	transcription factor binding		
		synaptic transmission	enzyme binding		
		axonogenesis	histone acetyltransferase binding		
		axon guidance	cAMP response element binding		

Gene Title	Gene Symbol	go biological process term	go molecular function term	go cellular component term	Pathway
		lactation	sequence-specific DNA binding		
		memory	transcription regulatory region DNA binding		
		circadian rhythm			
		regulation of cell size			
		fibroblast growth factor receptor signaling pathway			
		response to organic substance			
		negative regulation of transcription by competitive promoter binding			
		viral process			
		pituitary gland development			
		cell differentiation			
		mammary gland development			
		positive regulation of transforming growth factor beta3 production			
		secretory granule organization			
		response to glucagon			
		toll-like receptor 2 signaling pathway			
		toll-like receptor 3 signaling pathway			
		toll-like receptor 4 signaling pathway			
		toll-like receptor 5 signaling pathway			
		toll-like receptor 9 signaling pathway			

Gene Title	Gene Symbol	go biological process term	go molecular function term	go cellular component term	Pathway
		toll-like receptor 10 signaling pathway			
		TRIF-dependent toll-like receptor signaling pathway			
		Fc-epsilon receptor signaling pathway			
		toll-like receptor TLR1:TLR2 signaling pathway			
		toll-like receptor TLR6:TLR2 signaling pathway			
		positive regulation of multicellular organism growth			
		response to drug			
		regulation of circadian rhythm			
		regulation of apoptotic process			
		innate immune response			
		positive regulation of fat cell differentiation			
		positive regulation of osteoclast differentiation			
		positive regulation of transcription, DNA-templated			
		positive regulation of RNA polymerase II transcriptional preinitiation complex assembly			
		positive regulation of transcription from RNA polymerase II promoter			
		positive regulation of hormone secretion			
		positive regulation of lipid biosynthetic process			
		neurotrophin TRK receptor signaling pathway			
		phosphatidylinositol-mediated signaling			

Gene Title	Gene Symbol	go biological process term	go molecular function term	go cellular component term	Pathway
		rhythmic process			
		protein stabilization			
		stress-activated MAPK cascade			
		lung epithelium development			
		lung saccule development			
		Type I pneumocyte differentiation			
		cellular response to zinc ion			
		cellular response to growth factor stimulus			
protein phosphatase 1, regulatory (inhibitor) subunit 1B	PPP1R1B	response to amphetamine	protein kinase inhibitor activity	nucleus	
		transcription, DNA-templated	protein phosphatase inhibitor activity	cytoplasm	
		negative regulation of protein kinase activity	protein serine/threonine phosphatase inhibitor activity	cytosol	
		signal transduction	protein binding	neuronal cell body	
		negative regulation of female receptivity	protein phosphatase type 1 regulator activity		
		visual learning	D1 dopamine receptor binding		
		intracellular signal transduction	D2 dopamine receptor binding		
		negative regulation of catalytic activity	D3 dopamine receptor binding		
		regulation of catalytic activity	D4 dopamine receptor binding		

Gene Title	Gene Symbol	go biological process term	go molecular function term	go cellular component term	Pathway
		negative regulation of cyclin-dependent protein kinase activity	D5 dopamine receptor binding		
